# Interaction of *Mycobacterium tuberculosis* RshA and SigH Is Mediated by Salt Bridges

**DOI:** 10.1371/journal.pone.0043676

**Published:** 2012-08-24

**Authors:** Shiva Kumar, Suguna Badireddy, Kuntal Pal, Shikha Sharma, Chandni Arora, Saurabh K. Garg, Mohamed Suhail Alam, Pushpa Agrawal, Ganesh Srinivasan Anand, Kunchithapadam Swaminathan

**Affiliations:** 1 Department of Biological Sciences, National University of Singapore, Singapore, Singapore; 2 Institute of Microbial Technology, CSIR, Sector 39-A, Chandigarh, India; University of Padova, Italy

## Abstract

The alternate sigma factor *sigH* of *Mycobacterium tuberculosis* is expressed under stress and acts as a major regulator of several genes, including some other sigma factors and redox systems. While it is auto-regulated by its own promoter at the transcriptional level, its regulation at the post-translational level is through its cognate protein, an anti-sigma factor, RshA. Hither before RshA was believed to be a zinc-associated anti-sigma factor (ZAS) and the binding of RshA to SigH is redox dependent. Here, we show that RshA coordinates a [2Fe-2S] cluster using cysteines as ligands and native RshA has more affinity to [2Fe-2S] cluster than to zinc. Furthermore, we used amide hydrogen deuterium exchange mass spectrometry (HDX-MS), followed by site-directed mutagenesis in SigH and RshA, to elucidate the interaction mechanism of RshA and SigH and the potential role of metal ion clustering in SigH regulation. Three regions in SigH, comprising of residues 1–25, 58–69, 90–111, 115–132 and 157–196 and residues 35–57 of RshA show decreased deuterium exchange and reflect decreased solvent accessibility upon complexation with SigH. Of the three RshA mutants, created based on the HDX results, the RsHA E37A mutant shows stronger interaction with SigH, relative to WT RshA, while the H49A mutant abolishes interactions and the C(53)XXC(56)AXXA mutant has no effect on complexation with SigH. The D22A, D160A and E162 SigH mutants show significantly decreased binding to RshA and the E168A mutant completely abolished interactions with RshA, indicating that the SigH-RshA interaction is mediated by salt bridges. In addition, SigH-RshA interaction does not require clustering of metal ions. Based on our results, we propose a molecular model of the SigH-RshA interaction.

## Introduction

After its initial onset of infection in humans, the pathogen *Mycobacterium tuberculosis* (Mtb) becomes latent in a large number of cases, until a cellular stimulus reactivates it. During latency, gene expression is mostly regulated at the pre-transcription level, where sigma factors play a major role. Apart from major sigma factors, the bacterium also harbors alternate sigma factors or extracytoplasmic function sigma factors. They regulate gene expression, mainly when cells encounter extra or intracellular stress [1].

The alternate sigma factor *sigH* of Mtb is a master regulator and is expressed under heat shock, oxidative, nitrosative, acid stress and phagocytosis [2], [3], [4], [5], [6], [7]. A large number of genes that are involved in the survival of Mtb under oxidative stress and virulence are regulated by *sigH* [4], [8]. The expression of two alternate sigma factors, *sigE* and *sigB*, is also under the control of *sigH* [4], [9], [10], [11]. In addition, under oxidative stress, *sigH* induces the expression of Mtb redox systems, such as thioredoxins *trxA*, *trxB1*, *trxC* and thioredoxin reductase *trxB2*, whereas, it induces the expression of the *hsp70* and *clpB* genes under heat stress [4], [8], [12]. In the related organism *Streptomyces*, the SigH homolog has been shown to regulate glucose dependent cell differentiation and antibiotic production [13]. At the transcriptional level, *sigH* is auto-regulated by its own promoter but at the post-translational level, its regulation is through its cognate protein, an anti-sigma factor, RshA.

Activation of Mtb *sigH* is redox dependent. However Mtb SigH does not contain any cysteines and hence cannot respond to redox changes on its own. It is a known fact that sigma factors are bound with anti-sigma factors and the dissociation of anti-sigma factors is essential for its activation. Several anti-sigma factors also respond to redox changes in the cytoplasm to regulate the function of sigma factors. The Mtb anti-sigma factor RshA, encoded by the ORF Rv3221A, is located in the *sigH* operon and has been demonstrated to regulate the function of *sigH*/Rv3223c. The binding of RshA to SigH has been shown to be redox dependent [14], similar to RseA and SigE [15] and RsrA and SigR of *Streptomyces coelicolor* [16]. Some of the anti-sigma factors, like, RseA, RsaL and RsrA, have been shown to coordinate zinc and hence they are called zinc-associated anti-sigma factors (ZAS). They have a conserved HX_3_CX_2_C motif [17], [18], [19], [20]. However, RsmA of *Streptomyces coelicolor*, a HATPase_C family anti-sigma factor has a HX_3_CX_2_S motif and coordinates a [2Fe-2S] cluster. It has also been demonstrated that the interaction between RsmA and its associated sigma factor σ^M^ depends on the presence of a [2Fe-2S] cluster [21]. Therefore, the mere presence of a ZAS motif in a protein does not imply that the protein is capable of co-ordinating zinc. For example, Hsp33, which also binds Zn, has a CXCX_27–32_CX_2_C motif, where all four cysteine residues bind to a single Zn atom [22]. It is assumed that since Mtb RshA has a ZAS motif and its closest homologue RsrA of *S. coelicolor* is a zinc binding protein, where Zn ion plays a major role, RshA has also been proposed to be a zinc binding protein and its interaction with SigH is regulated by zinc. However, presence or binding of Zn with RshA or its direct involvement in mediating SigH and RshA interactions has not been clearly demonstrated so far.

Apart from metal ions, phosphorylation of both SigH and RshA, by PknB, alters their interaction. It has been shown that *in vivo* phosphorylation of RshA results in poor binding to SigH [23], which also means partial inhibition of SigH action. At the transcription level, SigH expression is auto-regulatory. Therefore, it is possible that under conditions where input stress signals are very strong, RshA is phosphorylated, thereby resulting in pools of free SigH that are capable of binding RNA polymerase. Formation of the holoenzyme would enable transcription and then SigH complexed with RNA polymerase would bind to its own promoter and regulate transcription [3]. Therefore, this could be an alternate mechanism of regulation of SigH in Mtb which might be important at some stages of its survival in its host.

Even though the functions of SigH and RshA are well understood, there are still many unanswered questions. A previous study [24] has used the phage display and surface plasmon resonance (SPR) techniques to map the interactions of RshA and SigH, and identified a few peptides in RshA and one of them reduced *sigH* mediated transcription. Li et al. [25] have shown how the homologs RsrA and SigR of *Streptomyces coelicolor* interact. However, no structural map of the RshA-SigH complex exists. Furthermore, it is unknown if the mechanism of RshA-SigH interactions are identical to that of the RsrA and SigR interaction and probe details on the importance of the metal ion cluster. Additionally, such studies could be put into a better perspective had the interaction been probed with intact proteins and not isolated peptides. To address these questions, we have mapped the interaction of the RsHA and SigH proteins by amide hydrogen deuterium exchange mass spectrometry (HDX-MS) to map the RshA-SigH interface and induced conformational changes resulting from disruption or formation of cysteine bonds in RshA during its transition between oxidized and reduced states.

## Materials and Methods

### Bacterial Strains and Reagents

Genomic DNA from *Mtb* H37Rv was prepared as described earlier [26]. *Escherichia coli* DH5α was used for general cloning procedures, whereas expression of recombinant proteins was carried out in the BL21 (DE3) strain. Standard recombinant DNA techniques were followed [27].

### Cloning of *sigH* and *rshA* and Site Directed Mutagenesis

The *sigH*/Rv3223c gene was amplified using the Forward 5′ ATA TGA ATT CAT GGC CGA CAT CGA TGG TGT AAC CG 3′ and Reverse 5′ ATA TCT CGA GTG ACG ACA CCC CCT CGT GCG CCT G 3′ primers containing *Eco*RI and *Xho*I restriction sites, respectively (underlined) and inserted into the pGEX4T-1 or pET29a vectors to generate either GST or 6×His-tagged proteins, respectively. The following SigH mutants were generated using the corresponding primer sets. **D22A**: Forward 5′ CCG TCT GAG GAG ACA GCT GAG GAG TTG ACC GC 3′, Reverse 5′ GC GGT CAA CTC CTC AGC TGT CTC CTC AGA CGG 3′; **D160A**: Forward 5′ GGT CTA CTA CGC CGC TGT CGA AGG TT 3′, Reverse 5′ AAC CTT CGA CAG CGG CGT AGT AGA CC 3′; **E162A**: Forward 5′ CCG ATG TCG CAG GTT TCC CCT AC 3′, Reverse 5′ GTA GGG GAA ACC TGC GAC ATC GG 3′; **E168A**: Forward 5′ TCC CCT ACA AGG CTA TCG CCG AG 3′, Reverse 5′ CTC GGC GAT AGC CTT GTA GGG GA 3′.

The 303 bp *rshA*/Rv3221A gene was PCR amplified using the Forward 5′ ATA TAT GAA TTC GTG AGC GAA AAT TGC GGT CCG AC 3′ and Reverse 5′ ATA TAT GTC GAC GGG CCC TCC ACG GAT GAT GGT GG 3′ to generate either a GST or 6×His-tagged primers containing the *Eco*RI and *Sal*I restriction sites (underlined) and inserted into the pGEX4T-1 or pET29a vector. The following RshA mutants were generated using the corresponding primers sets. **E37A**: Forward 5′ CCG GAA ACC CGC GCT AGG CTG CGG CGA CAC CTC 3′, Reverse 5′ GAG GTG TCG CCG CAG CCT AGC GCG GGT TTC CGG 3′; **CXXC to AXXA**: Forward 5′ CTC GAG GCC GCT CCG GGG GCT CTG AGG CAT 3′, Reverse 5′ ATG CCT CAG AGC CCC CGG AGC GGC CTC GAG 3′; **H49A**: Forward 5′ AGA GGC TGC GGC GAG CAC TCG AGG C 3′, Reverse 5′ GCC TCG AGT GCT CGC CGC AGC CTC T 3′. The authenticity of all clones was confirmed by sequencing both strands of DNA using either T7 promoter or gene specific primers.

### Protein Production

The proteins were overexpressed in the pET29a vector with an N-terminal S-tag and C-terminal 6xHis tag. BL21 (DE3) cells containing the overexpression constructs were grown on a shaker incubator (LB broth, 37°C, 30 µg/ml Kanamycin) until OD_600_ was ∼0.6–0.8. The culture was cooled to 16°C and induced with 300 µM IPTG and the cells were grown for 12 to 16 h. The cells were then harvested by centrifugation at 3000 g and either stored at −80°C as pellet for later use or re-suspended in buffer A (50 mM Tris (pH 8.0), 300 mM NaCl, 25 mM imidazole and 5 mM β-mercaptoethanol (BME)) for lysis in a French press at 10,000 psi. The cell lysate was then centrifuged at 39,000 g for 20 min to remove all cell debris and the supernatant was loaded onto a Ni chelating Sepharose column (10 ml resin in a 50 ml column). The resin was then washed with 600 ml buffer A to remove all unbound proteins. The bound protein was then eluted with 15 ml buffer B (50 mM Tris (pH 8.0), 300 mM NaCl, 250 mM imidazole and 5 mM BME). The elute was then concentrated and loaded onto an S75 column (GE), equilibrated with buffer C (50 mM Tris (pH 8.0), 300 mM NaCl and 5 mM DTT) and mounted on an AKTA FPLC system (GE), for size exclusion chromatography.

### Alkylation of RshA

Iodoacetamide, an irreversible inhibitor of cysteine peptidases, binds specifically to cysteine residues. Freshly purified RshA was dialyzed against buffer containing 50 mM Tris HCl (pH 7.5), 100 mM NaCl, 10 mM DTT for 15 h at 4°C with two changes, followed by incubation with 20 mM iodoacetamide for 60 min at 37°C in dark. Excess iodoacetamide was removed by dialysis in 50 mM Tris HCl (pH 7.5) and 100 mM NaCl, 10 mM DTT for 15 h at 4°C.

### Zn Saturation of RshA

Freshly purified RshA was dialyzed against buffer containing 50 mM Tris HCl (pH 7.5), 100 mM NaCl, 10 mM DTT for 15 hr at 4°C with two changes. The protein was incubated with a two molar excess of ZnCl_2_ for 30 min at 37°C. The Zn saturated protein was again dialyzed in 50 mM Tris HCl (pH 7.5), 100 mM NaCl, 1 mM DTT for 15 h at 4°C to remove excess Zn.

### Measurement of Zinc Content

10 µM RshA was incubated in 500 µl of 50 mM Tris HCl (pH 7.5), 100 mM NaCl with 40 µg proteinase K at 56°C for 30 min to release all Zn from the protein. The volume of reaction was increased with buffer to 994 µl, then 6 µl of 25 mM 4-(2-pyridylazo)-resorcinol (PAR) was added to the sample. The absorbance was measured at 500 nm on a Hitachi U2800 spectrophotometer. Zn estimation was carried out by comparing the absorbance with a standard curve that was prepared with known amounts of ZnCl_2_.

### Measurement of Iron Content

To measure the total iron content of RshA, the o-bathophenanthroline (OBP) method was used. To chelate the iron, OBP was added at a final concentration of 10 mM to the protein solution and the reaction was carried out in dark at 25°C for 1 h. Chelation was monitored by the increase in absorbance at 540 nm, using buffer alone as a control. The quantity of chelated iron was determined using an extinction coefficient of 22140 M^−1^cm^−1^. The absorbance was recorded on a λ35 PerkinElmer spectrophotometer. The measurements were carried out in duplicate. The data presented is an average of three independent protein preparations.

### Fe-S Reconstitution of RshA

Freshly purified RshA was dialyzed against buffer containing 50 mM Tris HCl (pH 7.5), 100 mM NaCl, 10 mM DTT for 15 hr at 4°C, with two changes of the buffer. The protein was incubated in a five-fold molar excess of FeCl_3_ and Na_2_S, at 22°C for 4 h. The reconstituted protein was dialyzed in 50 mM Tris HCl (pH 7.5), 100 mM NaCl, 10 mM DTT for 10 h at 4°C to remove all unbound Fe^3+^ and S^2−^. The protein was scanned (between 200 and 800 nm) on a PerkinElmer λ35 spectrophotometer at 25°C to show the presence of the Fe-S cluster.

### GST Pull-down Assay

Wild-type and mutant proteins were cloned into the pGEX4T-1 vector and were expressed using same protocol as described earlier. Soluble fractions of two cell lysates (5.0 mg crude protein each) were mixed together in buffer containing 50 mM Tris HCl (pH 7.5), 100 mM NaCl, 10 mM DTT and 0.01% Triton X-100 and allowed to interact at 4°C for 3 h on a rocker platform. DTT was periodically replenished during interaction. 3 mg glutathione resin (Sigma) was allowed to swell in the above buffer for 2 h and washed twice in the same buffer. The swollen beads were mixed with the lysates and incubated for 2 h at 22°C to facilitate binding of GST-fusion protein to the resin. Then the lysate was removed by micro-centrifugation at 2000 rpm for 2 min at 22°C and then was washed twice with the above buffer and once each with 50 mM Tris HCl (pH 7.5), 200 mM NaCl, 10 mM DTT, 0.01% Triton X-100 and 50 mM Tris HCl (pH 7.5), 300 mM NaCl,. The washed resin was mixed with SDS gel-loading buffer and the bound proteins were resolved on 15% SDS-PAGE and stained with Coomassie blue or processed for immunoblotting [28].

### Amide Exchange Mass Spectrometry

Both proteins were concentrated to 80 µM before the exchange reaction. HDX-MS of the free proteins and complex was then carried out. The complex was generated by mixing each protein with a 1.5 time molar excess of its binding partner protein (80 µM). As the K_d_ of the two proteins is approximately 15 nM [24], all binding sites were expected to be occupied during the exchange reaction. The complexes were mixed at the desired molar ratio and concentration and were incubated at 4 °C for 12–16 h prior to HDX-MS. 2 µl of the free proteins and complex were diluted with 18 µl of D_2_O containing 5 mM DTT (99.9%) to give a final deuterium concentration of 90% in buffer C. Exchange was carried out at 20°C for the time points: 0.5, 1, 2, 5 and 10 min and was quenched by addition of 40 µl of pre-chilled 0.1% trifluoroacetic acid (TFA) to yield a final pH_read_ of 2.5. 50 µl of the sample was then injected onto a chilled nanoUPLC sample manager (beta test version, Waters, Milford, MA) as previously described [29]. The sample was washed through a 2.1×30 mm immobilized pepsin column (Porozyme, ABI, Foster City, CA) using 100 µL/min 0.05% formic acid in water. Digested peptides were trapped on a 2.1×5 mm C18 trap (ACQUITY BEH C18 VanGuard pre-column, 1.7 µm resin, Waters, Milford, MA). The peptides were eluted using an 8–40% gradient of acetonitrile in 0.1% formic acid, which was supplied by a nanoACQUITY Binary Solvent Manager, at a flow rate of 40 µL/min onto a reverse phase column (Acquity UPLC BEH C18 column, 1.0×100 mm, 1.7 µm, Waters, Milford, MA). Peptides were detected and mass was measured on a Synapt HDMS mass spectrometer (Waters, Manchester, UK) acquiring in the MS^E^ mode, a non-biased, non-selective CID method [30], [31], [32], [33].

Sequence identifications were made from the MS^E^ data from undeuterated samples using ProteinLynx Global Server 2.4 (beta test version, Waters, Milford, MA) [33], [34] and searched against the sequences of RshA and SigH with no enzyme specified and no modifications of amino acids. Identifications were only considered if they appeared at least twice out of three replicate runs. The precursor ion mass tolerance was set at <10 ppm and fragment ion tolerance was set at <20 ppm. Only those peptides that satisfied the above criteria through Database search pass 1 were selected. The default criteria for false positive identification (Value = 4) was applied. These identifications were mapped to subsequent deuteration experiments using prototype custom software (HDX browser, Waters, Milford). Centroid values for each peptide at all time points were extracted using this software, and exported to HX-Express [35] for analysis. A total number of 34 and 31 fragments yielded primary sequence coverage of 71 and 88% for SigH and RshA, respectively. The average number of deuterons exchanged in each peptide was calculated by subtracting the centroid value for the un-deuterated peptide from the centroid for deuterium exchanged peptides at each time point determined.

Continuous instrument calibration was carried out with Glu-fibrinogen peptide at 100 fmol/µL. A control experiment was carried out to calculate the deuterium back exchange loss using the ligand-free cAMP dependent protein kinase regulatory subunit as a model protein as described previously, yielding a deuterium back exchange value of 32.7% [36]. All reported deuterium exchange values were corrected accordingly.

## Results

### RshA Coordinates a [Fe-S] Cluster

**Figure 1:**
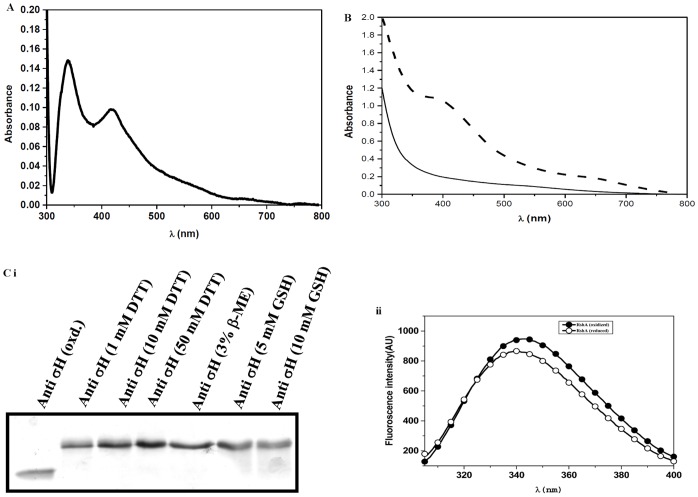
Biophysical characterization of RshA. (**A**) The absorption scan of freshly purified RshA shows two characteristics peaks at 340 and 420 nm, respectively, confirming the presence of a [2Fe-2S] cluster. (**B**) [Fe-S] binding property, after reconstitution, of non-alkylated (dashed line) and alkylated (solid line) RshA. The peak at 410 nm for the non-alkylated RshA protein suggests the presence of a [4Fe-4S] cluster. Alkylation abolishes the metal binding property of RshA. (**C**) Conformational changes in RshA due to reduction of disulfide bonds. (**i**) Purified RshA was treated with different reducing agents for 2 h at 25°C. (**ii**) Intrinsic tryptophan fluorescence of oxidized and reduced RshA.

Freshly purified recombinant RshA protein (101 aa) was brown in color (Fig. S1). A spectral scan (between 200–800 nm) showed two distinct peaks at 340 and 420 nm (Fig. 1A), the characteristic feature of an [Fe-S] cluster, suggesting that RshA coordinates an [Fe-S] cluster. The iron content of the freshly purified protein was sub-stoichiometric but the [Fe-S] cluster reconstituted protein had an iron content of 3.235±0.552 atoms per molecule of RshA (Table 1), an indicator that RshA coordinates a [4Fe-4S] cluster. The drop in the iron content can be attributed to the fact that the [4Fe-4S] cluster of RshA is highly unstable. As we could not analyze the [Fe-S] cluster by electro-paramagnetic resonance (EPR), it is not possible to comment on the oxidation state of the [Fe-S] cluster of RshA.

**Table 1:** Estimation of iron in RshA.

Sample	Number of iron atom per RshA
Freshly purified RshA	0.117±0.033
Oxidized and dialyzed RshA	0.009±0.0003
Fe-S reconstituted RshA	3.235±0.552
Alkylated and Fe-S reconstituted RshA	0.236±0.0932

Iodoacetamide (IAA) alkylates cysteine residues and alkylated cysteines cannot coordinate any metal ion. While un-alkyalted RshA coordinated an [Fe-S] cluster, alkylated RshA did not coordinate an [Fe-S] cluster, Fig. 1B. This suggests that cysteines are the ligands for [Fe-S] cluster coordination. It must be noted that RsmA from *Streptomyces coelicolor*, a putative member of the HATPase_c family of anti-sigma factors, also coordinates an [2Fe-2S] cluster [21] and the binding of RsmA to σ^M^ is dependent on the presence of an [Fe-S] cluster.

### RshA has Weak Affinity for Zinc

Since the current understanding is that RshA is a Zn coordinating protein (due to the presence of a HX_3_CX_2_C motif), we measured the zinc coordinating properties of RshA using the 4-(2-pyridylazo)-resorcinol (PAR) method (Table 2). The total Zn content of RshA was measured immediately after protein purification and also after dialysis, which removed all salt. In either of the conditions we did not find any Zn in the protein. Assuming that zinc ions of the protein were leached during purification and dialysis, purified RshA was saturated with zinc and the total zinc content of the protein was measured. The result showed that each RshA molecule would coordinate ∼0.750±0.110 atoms of Zn. To confirm that RshA indeed has poor affinity for zinc compared to iron, we attempted to displace the bound iron with zinc. The experiment was based on the premise that if RshA is a zinc binding protein then it will have higher affinity to zinc than iron and zinc would be able to displace the [Fe-S] cluster. Therefore, RshA was saturated with [Fe-S] cluster and after removing all excess iron by dialysis, ZnCl_2_ was added in equimolar and two molar concentrations. The change in the spectral properties of the protein was monitored at different time intervals, before and after dialysis. Fig. S2 clearly shows that zinc could not displace the bound iron from RshA, suggesting that RshA has higher affinity for iron than Zn. However, in a reverse experiment, the [Fe-S] cluster could easily replace zinc, Fig. S2(b). These results clearly demonstrate that RshA has a higher affinity for [Fe-S] than to zinc.

**Table 2:** Estimation of zinc in RshA.

Sample	Number of zinc atom per RshA
Freshly purified RshA	0.052±0.011
Oxidized and dialyzed RshA	0.009±0.0005
Zinc saturated RshA	0.750±0.110
Alkylated and zinc saturated RshA	0.662±0.063

RshA has 6 cysteine residues where five are conserved amongst anti-sigma factors. Our results show that RshA undergoes significant conformational change after reduction, as evidenced by SDS-PAGE and intrinsic tryptophan fluorescence of both oxidized and reduced RshA, Fig. 1C(ii). This suggests that the cysteines in RshA form disulfide bonds, which would have major conformational role in RshA.

### SigH Undergoes Huge Conformational Changes Upon Binding with RshA

**Figure 2:**
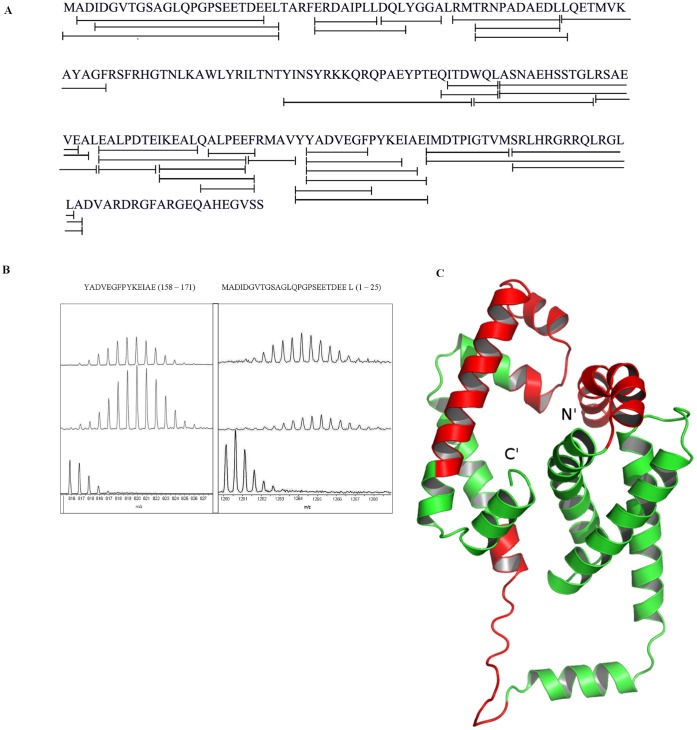
HDX-MS of SigH. (**A**) Sequence coverage map for SigH. Solid line denotes the pepsin digest fragments analyzed in the study with a total sequence coverage of 71%. (**B**) ESI-Q-TOF mass spectra for 2 pepsin digest fragments of SigH, (i) (157–171); m/z = 815.89, z = 2 and (ii)* (1–25); m/z = 1259.56, z = 2 which showed significant differences in exchange upon RshA binding. (I) Undeuterated peptide (II) The isotopic envelope for the same peptide from free SigH following 10 min deuteration; (III) The isotopic envelope for the same peptide from SigH and RshA complex following 10 min deuteration. *- The default display of the isotopic envelope for this peptide (1–25); m/z = 1259.56 from the mass spectrometry program, HDX Browser was in color. For clearer display, spectra were highlighted in black (**C**) The SigH model was prepared using the program I-Tasser using the homologous structure of SigR from *Streptomyces coelicolor*. The protein is shown in green. The regions in red represent regions of the SigH protein showing decreased deuterium exchange in the presence of its interacting partner, RshA.

To probe the interactions between RshA and SigH, we used HDX-MS for free RshA, SigH proteins and the RshA-SigH complex. A total of 32 pepsin digest fragments peptides common to free SigH and SigH in complex with RshA were identified and analyzed. These 32 pepsin digest fragments constitute a primary sequence coverage of ∼71% (Fig. 2A). Deuterium exchange (t = 10 min) for these peptides is tabulated in Table 3.

**Table 3:** Summary of amide H/D exchange in pepsin digest fragments from SigH (Deuterium exchange time = 10 min)^a^.

No.	Pepsin digest fragment	Peptide, m/z, Charge state	SigH in complex with RshA	SigH
1	1–25	MADIDGVTGSAGLQPGPSEETDEEL (1259.56), +2	9.3±0.1	12.0±0.1
2	2–23	ADIDGVTGSAGLQPGPSEETDE (1072.980), +2	6.9±0.1	8.2±0.2
3	4–25	IDGVTGSAGLQPGPSEETDEEL (1101.01),+2	9.2±0.1	11.3±0.0
4	30–40	ERDAIPLLDQL (641.86), +2	5.2±0.0	6.0±0.2
5	38–44	DQLYGGA (723.331), +1	4.1±0.0	4.2±0.0
6	46–57	RMTRNPADAEDL (694.84), +2	6.6±0.1	6.9±0.1
7	48–57	TRNPADAEDL (1101.52), +1	5.2±0.0	5.6±0.1
8	48–58	TRNPADAEDLL (1214.60), +1	5.4±0.0	6.1±0.2
9	58–69	LQETMVKAYAGF (679.35), +2	6.5±0.1	7.7±0.4
10	90–111	YINSYRKKQRQPAEYPTEQITD (910.12), +3	7.8±0.3	10.0±0.0
11	108–114	QITDWQL (903.46), +1	2.4±0.1	2.7±0.0
12	109–114	ITDWQL (775.40), +1	1.6±0.0	1.9±0.0
13	112–125	WQLASNAEHSSTGL (750.86), +2	3.9±0.1	4.6±0.0
14	115–131	ASNAEHSSTGLRSAEVE (872.91), +2	4.8±0.2	6.0±0.2
15	115–132	ASNAEHSSTGLRSAEVEA (908.43), +2	5.4±0.1	7.3±0.0
16	126–133	RSAEVEAL (874.47), +1	3.3±0.1	3.7±0.0
17	133–140	LEALPDTE (887.44), +1	3.1±0.1	2.8±0.0
18	134–145	EALPDTEIKEAL (664.852), +2	5.6±0.1	6.5±0.0
19	141–151	IKEALQALPEE (620.84), +2	5.7±0.1	6.5±0.0
20	141–152	IKEALQALPEEF (694.38), +2	6.1±0.1	6.8±0.1
21	144–152	ALQALPEEF (1017.53), +1	4.0±0.1	4.6±0.0
22	146–152	QALPEEF (833.40), +1	2.7±0.0	2.8±0.0
23	152–157	FRMAVY (786.40), +1	3.5±0.1	3.5±0.1
24	157–164	YYADVEGF (963.42), +2	4.5±0.2	5.2±0.0
25	157–171	YYADVEGFPYKEIAE (897.418), +2	8.2±0.2	9.4±0.1
27	158–168	YADVEGFPYKE (659.31), +2	5.2±0.1	5.7±0.0
28	158–170	YADVEGFPYKEIA (751.366), +2	7.2±0.2	8.1±0.0
29	158–171	YADVEGFPYKEIAE (815.89), +2	7.2±0.2	8.1±0.0
30	172–181	IMDTPIGTVM (1077.53),+1	3.9±0.1	4.4±0.0
31	182–195	SRLHRGRRQLRGLL (859.53), +2	4.2±0.1	4.8±0.0
32	182–196	SRLHRGRRQLRGLLA (895.046), +2	4.8±0.4	5.7±0.1

a Average number of deuterons exchanged determined following 10-min deuterium exchange. Values reported are the mean and standard deviation from at least two independent experiments.

Almost all of SigH with the exception of two regions, showed decreased exchange upon complexation with RshA. Among these peptides, the ones that display a change of >1 Deuteron exchange correspond to residues (1–25), (58–69), (90–111), (115–132), (157–171) and (172–196) (Table 3). Mass spectral isotope envelopes for two peptides, 1–25 and 158–171 are shown in Fig. 2B. This figure shows overlaid the isotopic envelope for the un-deuterated peptide fragment (bottom panel), deuterium exchange (t = 10 min) for SigH (middle panel) and SigH-RshA complex (top panel). There is a smaller shift to the right for the deuterium exchanged samples from the SigH-RshA complex compared to SigH alone. These peptides were mapped on to a homology model of SigH, which was obtained from the I-Tasser server [37], [38], [39], (Fig. 2C). The peptide (30–40) that is expected to form a hydrophobic core in SigH, based on homology with the available Sigma factor structures (PDB entries 1OR7, 2H27 and 1H3L) [25], [40], [41] showed decreased exchange. As a hydrophobic core region, it is unlikely to participate in interface formation with RshA. Decreased exchange therefore is indicative of increased order upon complex formation.

### Region 34–57 of RshA Showed Decreased Exchange Upon Binding to SigH

**Figure 3:**
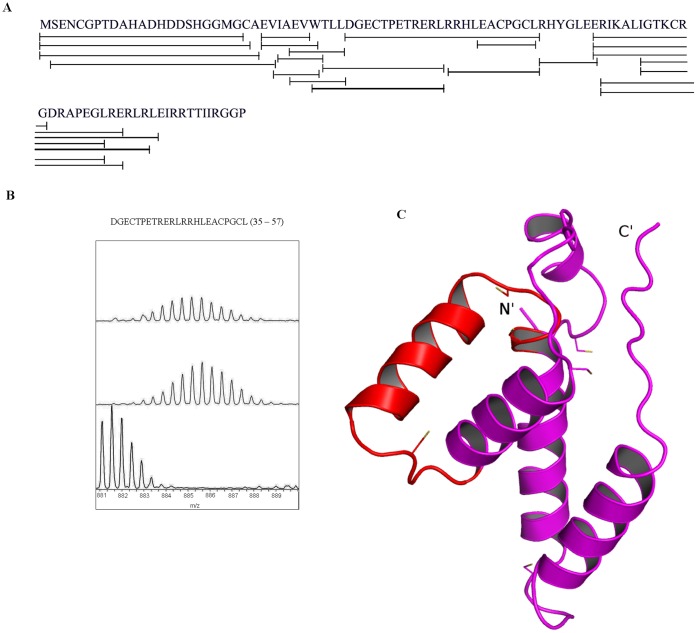
HDX-MS of RshA. (**A**) Sequence coverage map for RshA. Solid line denotes the peptic fragments analyzed in the study with total sequence coverage of 88%. (**B**) ESI-Q-TOF mass spectra for one pepsin digest fragment of RshA (35–57) m/z = 881.084, z = 3, which showed significant difference upon RshA binding. (i) Undeuterated RshA peptide (ii) The isotopic envelop for the same peptide from free RshA following 10min deuteration; (iii) The isotopic envelope for the same peptide from RshA and SigH complex following 10 min deuteration., The isotopic envelope for the same peptide. (**C**) The protein is shown in magenta. The region in red represents regions showing decreased exchange upon interactions with its partner, SigH.

A total of 20 pepsin digest fragment peptides common between free RshA and RshA in complex with SigH were identified and analyzed. These 20 pepsin digest fragment peptides constitute primary sequence coverage of ∼88% (Fig. 3A). Deuterium exchange (t = 10 min) for each of these peptides is tabulated in Table 4. HDX-MS revealed that one region with three overlapping peptides (34–49) (35–57) (Fig. 3B) and (47–57) showed decreased exchange upon binding with SigH. These peptides are mapped on to a homology model of RshA and shown in Fig. 3C. This region contain the consensus CXXC sequence, which is predicted to bind a metal ion cluster, which, in turn, is expected to form a direct interface for the interaction of RshA with SigH. However, contrary to our expectation, the CXXC to AXXA mutant of RshA did not affect binding to SigH, *vide infra*. In addition, overlapping peptides, (25–31), (26–31) (70–83) and (70–88), from two regions on RshA showed increased exchange in complex with SigH. This increased exchange might be caused by domain movement induced by SigH binding to RshA.

**Table 4:** Summary of amide H/D exchange in pepsin digest fragments from RshA (Deuterium exchange time = 10 min)^a^.

No.	Pepsin digest fragment	Peptide, m/z, Charge state	RshA in complex with SigH	RshA_
1	2–26	SENCGPTDAHADHDDSHGGMGCAEV (837.98), +3	7.8±0.1	8.2±0.1
2	25–30	EVIAEV (659.353), +1	3.1±0.1	2.8±0.0
3	25–31	EVIAEVW (845.437), +1	4.4±0.0	3.9±0.0
4	26–31	VIAEVW (716.396), +1	3.7±0.0	2.8±0.1
5	29–34	EVWTLL (760.422), +1	3.6±0.2	3.7±0.0
6	27–31	IAEVW (617.33), +1	2.5±0.0	2.1±0.1
7	30–34	VWTLL (631.378), +1	2.9±0.0	2.8±0.0
8	31–46	WTLLDGECTPETRERL (640.315), +3	10.2±0.1	10.1±0.1
9	32–46	TLLDGECTPETRERL (866.927), +2	10.0±0.0	9.7±0.1
10	34–49	LDGECTPETRERLRRH (656.664), +3	8.6±0.4	10.2±0.7
11	35–57	DGECTPETRERLRRHLEACPGCL (881.084), +3	13.1±0.0	15.0±0.1
12	47–57	RRHLEACPGCL (627.815), +2	4.1±0.2	4.8±0.0
13	51–57	EACPGCL (692.268),+1	2.7±0.0	2.8±0.2
14	58–64	RHYGLEE (903.422), +1	2.7±0.0	3.2±0.0
15	64–76	ERIKALIGTKCRG (722.919), +2	8.5±0.0	9.2±0.0
16	64–85	ERIKALIGTKCRGDRAPEGLRE (823.452), +3	13.4±0.1	13.5±0.0
17	65–83	RIKALIGTKCRGDRAPEGL (685.389), +3	10.2±0.1	10.2±0.0
18	65–85	RIKALIGTKCRGDRAPEGLRE (780.439), +3	12.1±0.1	11.7±0.1
19	70–83	IGTKCRGDRAPEGL (736.884), +2	6.5±0.1	5.7±0.1
20	70–88	IGTKCRGDRAPEGLRERLR (728.407), +3	11.2±0.0	11.8±0.0

a Average number of deuterons exchanged determined following 10-min deuterium exchange. Values reported are the mean and standard deviation from at least two independent experiments.

### Selected Mutations in RshA and SigH Affect Protein-protein Interaction

**Figure 4:**
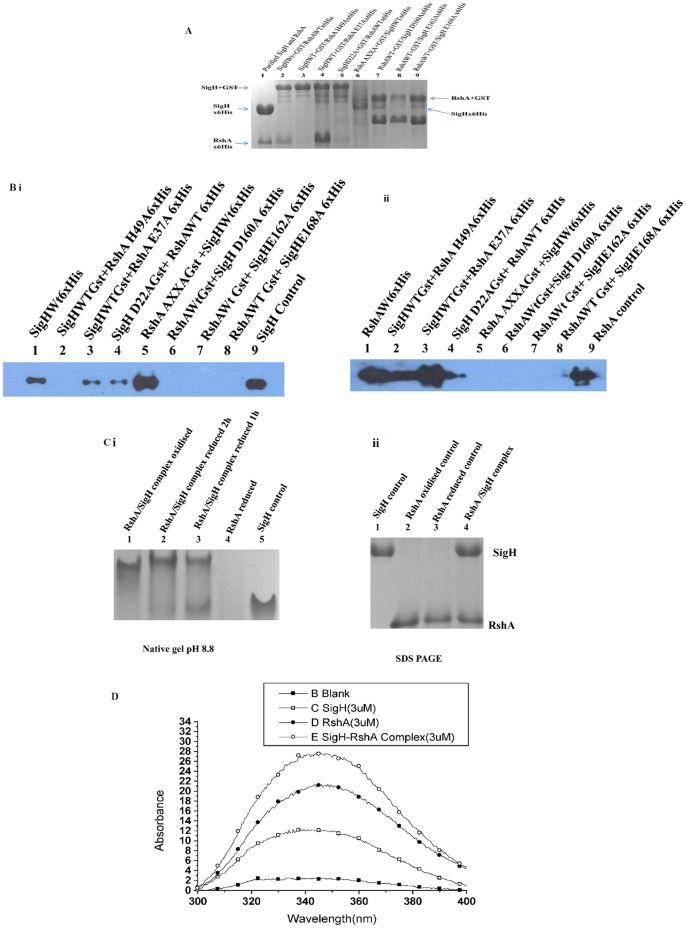
Targeted mutagenesis based on HDX-MS. Soluble fractions of two cell lysates (5.0 mg crude protein each RshA and mutants/SigH and mutants) were mixed together in a buffer containing 50 mM Tris HCl (pH 7.5), 100 mM NaCl, 10 mM DTT and 0.01% X-100 and allowed to interact at 4°C for 3 h on a rocker platform. DTT was periodically replenished during interaction. After interaction the protein mix was mixed with pre-swollen glutathione beads for 2 h at 22°C and then the beads were washed once each with 50 mM Tris HCl (pH 7.5), 10 mM DTT, 0.01% Triton X-100, 100 mM NaCl; 10 mM DTT, 0.01% Triton X-100, 50 mM Tris HCl (pH 7.5), 200 mM NaCl; 50 mM Tris HCl (pH 7.5), 300 mM NaCl; 10 mM DTT, 0.01% Triton X-100. The washed resin was mixed with SDS gel-loading buffer and the bound proteins were resolved on 15% SDS-PAGE and stained with Coomassie blue or processed for immunoblotting. (**A**) SDS-PAGE gel of a GST-pull down assay showing the interaction of WT RshA and WT SigH and their mutants. The gel was stained with Coomassie blue and another identical gel was used for Western Blot using anti 6xHis antibody. (**B**) Western blot using a 6xHis antibody in a GST-pull down assay to study the interaction of SigH and RshA. Both panel b and C have identical loading, however panel B represents SigH and and its mutants while panel C represents RshA and its mutants. (**i**) WT or mutant GST:RshA and 6xHis:SigH proteins were used. (**ii**) In the lower panel, WT or mutant GST:SigH and 6xHis:RshA proteins were used. (**C**) RshA-SigH interaction does not require metal ion. Purified 5 µM RshA/SigH each was mixed in the presence of 50 mM Tris HCl (pH 8.0), 100 mM NaCl, 0.01% Triton X-100 at 25°C. Same samples were loaded in both the native gel (ph 8.8) and SDS-PAGE and stained with Coomassie blue. The left panel is native gel and the right panel is SDS-PAGE. (**D**) Intrinsic tryptophan fluorescence of RshA (oxidized), SigH and their complex. 3 µM of each protein was mixed in a buffer containing 50 mM Tris HCl (pH 8.0), 100 mM NaCl, 0.01% Triton X-100 at 25°C for 2 hr to make the SigH-RshA complex. RshA has a single tryptophan while SigH has two tryptophan residues. The differences in the spectra clearly show that the complex formation is a dynamic process.

Based on our HDX results, three RshA mutants, E37A, H49A and CXXC to AXXA, were generated and their interactions with WT SigH were analyzed (Figs. 4A and 4B). The RshA E37A mutant interacted better with SigH than RshAWT, while the RshA H49A mutant had some negative effect. However, the AXXA mutant had no significant impact on the interaction. On the other hand, the SigH D22A, D160A and E162A mutants had some adverse effect on the interaction and the most important mutant is SigH E168A, which abolished the SigH-RshA interaction entirely.

The interaction result of the CXXC to AXXA mutant was surprising. It has been assumed that the CXXC motif might play a major role in promoting the RshA-SigH interaction as the CXXC motif is part of the ZAS (HXXXCXXC), a metal binding motif. Our results reveal that the AXXA mutant of RshA did not affect the interaction with SigH. This result raises a next question of whether [Fe-S] free RshA would interact with SigH. Metal free RshA (confirmed by spectrophotometry) was mixed with SigH at equimolar concentrations (3 µM each) for different times. The complexation reaction was then resolved on native gel as well as in SDS PAGE, Fig. 4A and C. Surprisingly, the native gel showed evidence of a complex, suggesting SigH-RshA interaction occurred even without the [Fe-S] cluster. It is to be noted that Mtb SigH does not have any cysteine residue. Therefore, unless there is an interface, RshA will not interact with SigH. We further confirmed the interaction of RshA and SigH in solution by monitoring changes in intrinsic tryptophan fluorescence in the RshA-SigH complex compared to unbound RshA and SigH. The fluorescence spectra in Fig. 4D clearly showed that while the emission maxima for SigH is 335 nm and that for RshA is 342 nm whereas the emission maxima of the complex is 345 nm. Peak broadening in the complex is also indicative of the RshA-SigH complex being a more dynamic complex. Furthermore, the result indicates that RshA interactions with SigH do not require metal ion. This result is in congruence with our GST-pull down assay where AXXA mutant of RshA showed no differences in interactions with SigH relative to wt-RshA.

## Discussion

The *Mycobacterium tuberculosis* stress response sigma factor SigH is a global regulator and also responds to heat shock. It is regulated by its cognate protein anti-sigma factor RshA. Similar to many proteins with a HX_3_CX_2_C motif, which binds a Zn atom, RshA was also proposed to be a Zn binding protein [14]. However, in our Zn saturation experiments, each RshA molecule bound to only ∼0.750 Zn atom. Surprisingly, RshA has not been investigated in detail compared to RsrA of *Streptomyces coelicolor* [42]. The *E. coli* cells that expressed RshA were brown in color and the cell lysate was also brown. Therefore, we attempted to investigate whether RshA would coordinate other metal ions, apart from Zn. Contrary to what is assumed in the scientific literature, our results showed that RshA is an [Fe-S] cluster coordinating protein, similar to Mtb RsmA [21]. Our attempt to replace the [Fe-S] cluster with Zn was unsuccessful, suggesting that RshA has lower affinity for Zn than iron. Furthermore, the [Fe-S] cluster of RshA could be reconstituted directly using inorganic iron and sulfur sources as well as by using cysteine desulfurase (Isc/Rv 2815c, cloned, expressed and purified (data not shown)). Furthermore, an [Fe-S] cluster will enable responses at a much faster rate to oxidative/reductive stress than Zn. SigH being a global regulator of Mtb, its quick response to stress will have a major implication for Mtb function. It is worthwhile to mention here that the [Fe-S] cluster of RshA is extremely sensitive to aerobic conditions and dissociates very quickly.

Iron-sulphur coordinating proteins are ubiquitous in nature. The [Fe-S] cluster is found in diverse families of proteins and is known to regulate the function of several transcription factors. Iron-sulfur clusters are key to the regulatory function of at least three transcription factors SoxR, IscR and FNR whereas, the function of OxyR, Spx, Hsp33, RsrA, Yap1 are directly or indirectly regulated by thiol-disulfide [43], [44], [45], [46], [47], [48]. Unlike SoxR, IscR and FNR, where an [Fe-S] cluster is needed for regulation, for aconitase, loss of the [Fe-S] cluster is essential before it binds to RNA [49]. Human mitochondrial glutaredoxin 2 (Grx2) was also reported to have an EPR silent non-oxidizable [2Fe-2S]^2+^ cluster that bridges two Grx2 molecules via two structural Cys residues to form dimeric holo-Grx2 [50]. Similar to aconitase, human holo Grx2 with an [Fe-S] cluster is enzymatically inactive and loss of the [Fe-S] cluster activates it.

From our HDX-MS studies, we conclude that only one region, spanning residues 35–57, in RshA exhibits decreased exchange in the RshA-SigH complex. Comparison of deuterium exchange in peptides 31–46, 34–49, 47–57 and 51–57 indicates that amino acids proximal to the N-terminus of motif CXXC showed decreased exchange in the complex, while the motif itself (residues 51–57) did not show any differences. These results highlight the importance of the regions flanking the metal ion cluster in RshA in mediating complexation with SigH, in which the metal ion itself (co-ordinated at CXXC) might be expendable, *vide infra*.

**Figure 5:**
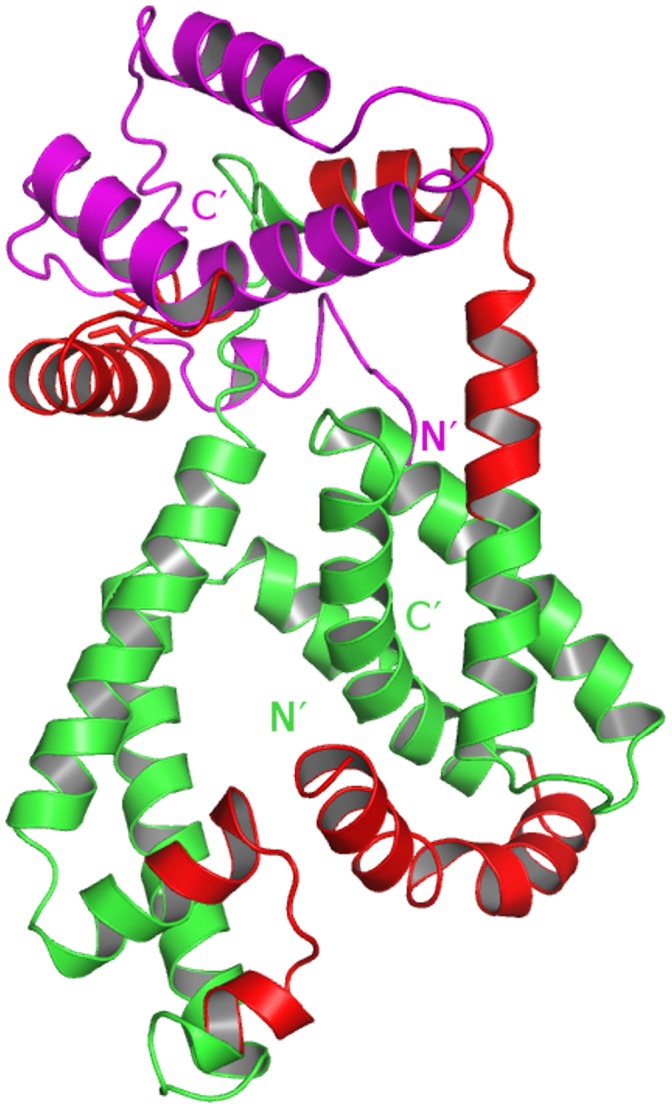
Model of interaction. A model for the interaction between SigH and RshA is proposed through a docking model, which was prepared with the help of the program Z-Dock [52]. The proteins follow the respective coloring schemes, as in Figs. 2d and 3d.

Three regions in SigH (residues 1–25, 90–132 and 157–196) showed decreased exchange upon complexation with RshA. Peptides spanning residues 1–25 and 157–196 show high homology with two available sigma factor structures (PDB entries 1H2L and 2H27). The central region in the amino acid sequence has low homology with available structures resulting in an increased dependence on *ab-initio* modelling to predict the relative orientation of the N and C terminal regions. Large regions of SigH display decreased exchange suggesting extensive domain rearrangements occurring while binding with RshA. Among these, the regions which display largest magnitude decreases in deuterium exchange are mapped onto the structural of SigH (Fig. 2C) and the docking model (Fig. 5). Our experimental results, summarized using the derived docking model, suggest that RshA might interact in the central region of SigH causing the N and C terminal regions to come together in an auto-inhibitory conformation which might shield access to other protein molecules. Higher magnitude decreases in exchange in the N and C terminal regions are therefore a likely result of domain movement due to the binding of RshA. These in turn might affect SigH’s ability to interact with other proteins and prevent transcription initiation. In addition, our results indicate that SigH might be a partially unstructured protein in the apo form, but, gets ordered upon complexation with RshA. We have observed similar ordering due to conformational selection in other studies on the cAMP-binding regulatory subunit (RIα) of Protein Kinase A [36].

Mutations in both RshA and SigH were designed based on the HDX –MS results and sequence homology to localize the amino acids important for formation of the RshA-SigH complex.. None of the three mutants in RshA, E37A, H49A and AXXA, abolished the interaction with SigH but H49A mutation had some adverse effect, indicating that a histidine at this position has some role in RshA/SigH interaction. Song et al. [14] have shown that single cysteine mutations reduce the binding of RshA with SigH. They postulated that mutation of cysteines to alanines should lock RshA in a reduced conformation, and therefore, aid in interaction with SigH if the interaction is only due to the conformational changes that could occur during the formation/disruption of disulfide linkages in redox conditions. However, we do not see any loss of interaction for the RshA CXXC AXXA mutant and this might indicate that cysteines of the CXXC motif of RshA do not form a disulfide bond, as shown in RsrA [51]. Thus we propose a more complex role for the metal ion cluster. We hypothesize that if an interaction with the metal ion cluster, as opposed to disulfide bond disruption, should drive the RshA interaction with SigH, then a large magnitude decrease in deuterium exchange upon complexation in this region should be expected. Our HDX results support such a model.

Based on the HDX results, the E37A mutant of RshA was generated but GST pull-down assay showed that the RshA E37A mutant does not affect the SigH-RshA interaction. The C(53)XXC(56) motif of RshA is at the end of a peptide that interacts with SigH. However, the CXXC to AXXA mutation did not disrupt the interaction. Also, even though the H49A mutant did not completely interrupt the interaction but it did inhibit the interaction to some extent. Zdanowski et al. [51] have shown that RsrA His37 (homologous position for His49 of RshA) has an important role in *vivo*. Further mutational analysis of RsrA has shown that His37, Cys41, Cys44 and Phe38 affected the interaction with SigR by apparently affecting the structural integrity of RsrA [42]. In SigH, while the D22A, D160A and E162A mutations adversely affected the interaction, the SigH E168A mutant did not interact with RshA. The RshA and SigH interaction takes place even in the absence of the metal cluster and also at multiple sites. These results prompt us to conclude that the SigH-RshA interaction is simply mediated by salt bridges and neither the cysteine residues nor the metal cluster has any significant role.

Based on these results, we propose a molecular model of the SigH-RshA interaction, Fig. 5. Using structures of homologous proteins and guided by our HDX and mutational analysis, we have modeled the interaction interface of SigH and RshA as indicated. This is a first step towards unraveling molecular details of this important protein interface. The interaction between SigH and RshA is redox dependent and hence any *in vitro* study would have to be carried out under reducing conditions. Furthermore, the interaction is stoichiometric. A 3-dimensional structure of this complex will provide a higher resolution view of the complex and provide insights into mycobacterial research as SigH regulates a large number of genes and several other Sigma factors. The structural and molecular determinants of the Mtb SigH and RshA interaction will be pivotal to understand the pathogen’s adaptation in host and virulence. The ability to specifically disrupt this interaction promises a potential therapeutic strategy against the pathogen’s defense within the host during the latent/asymptomatic phase.

## Supporting Information

Figure S1 **RshA protein production.** (A) SDS-PAGE gel, stained with Coomassie blue, of induced and uninduced cell samples, along with the purified RshA protein and a molecular weight marker. (B) The RshA protein is naturally brown in color, right tube, confirming its inherent property of iron binding. The left tube is buffer, as a color control.
(TIF)

Figure S2 **Displacement of Zn and Fe-S.** (A) Zn atom could not displace the Fe-S cluster. Fifty µM RshA was reconstituted with the [Fe-S] cluster and then treated with equimolar and two molar excess of ZnCl_2_ for different time periods to estimate the affinity of protein with both the metals. % residual [Fe-S] cluster is based on the A_400_. (B) Displacement of zinc by [Fe-S] cluster in zinc saturated RshA protein. 50 µM RshA was saturated with Zn and then subjected for reconstitution with equimolar concentrations of Fe^3+^ and sulfide. The absorption scan profile confirms the presence of a [4Fe-4S] cluster and [Fe-S] could easily replace zinc. Note that RshA has more affinity to [Fe-S] than to zinc. All the absorption scans were observed between 300 and 800 nm.
(TIF)
