# Supplementary figures and images for: Interaction of Mycobacterium tuberculosis RshA and SigH Is Mediated by Salt Bridges
Source: PLoS One. 2012 Aug 24;7(8):e43676. doi: 10.1371/journal.pone.0043676 (PMC3427169; doi:10.1371/journal.pone.0043676)

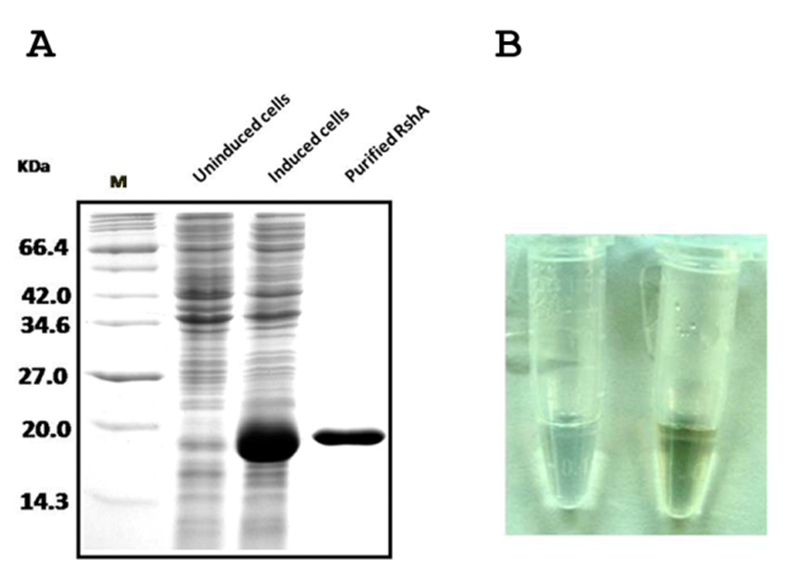

Supplement: Figure S1 — RshA protein production. (A) SDS-PAGE gel, stained with Coomassie blue, of induced and uninduced cell samples, along with the purified RshA protein and a molecular weight marker. (B) The RshA protein is naturally brown in color, right tube, confirming its inherent property of iron binding. The left tube is buffer, as a color control. (TIF) [file pone.0043676.s001.tif]

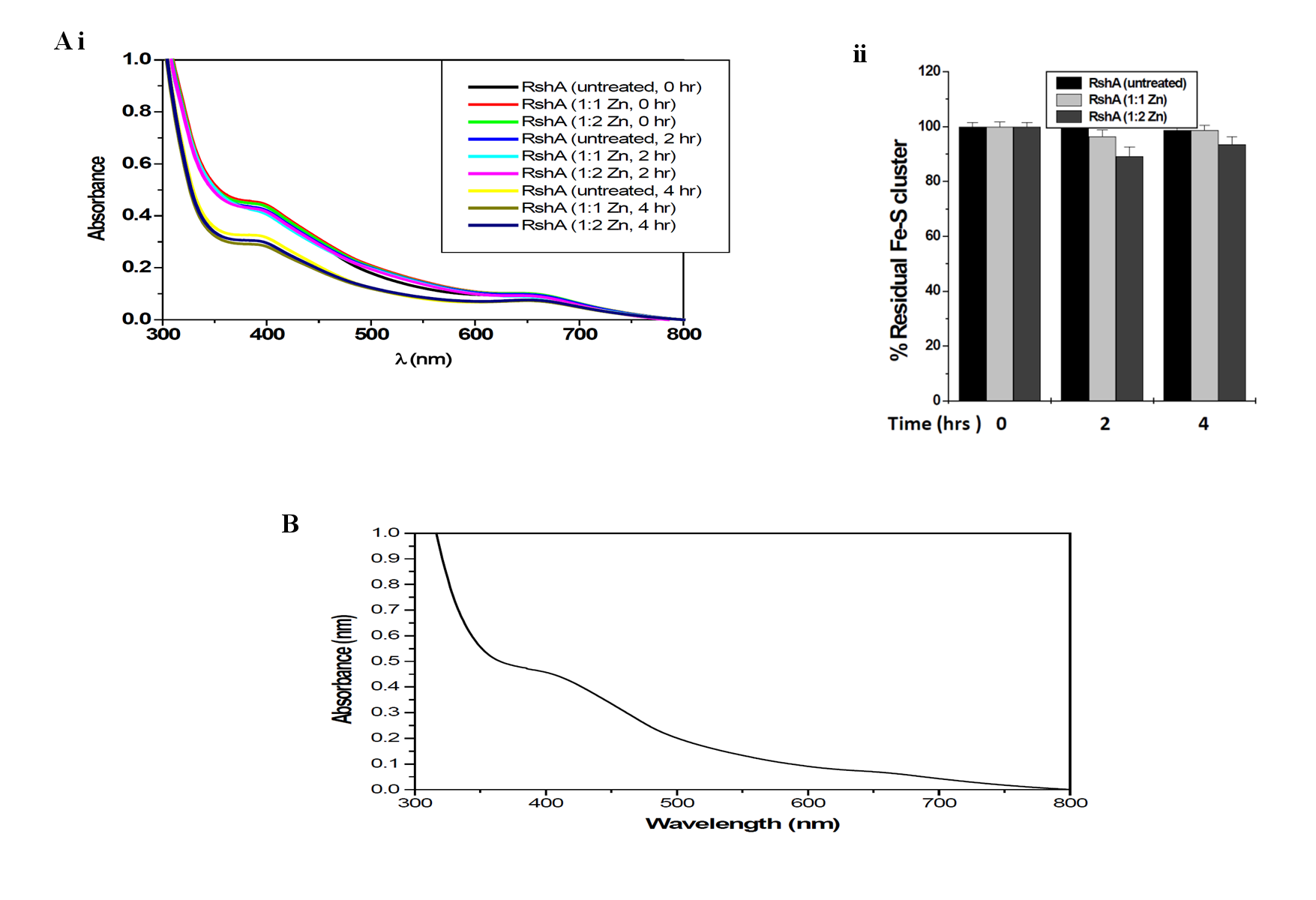

Supplement: Figure S2 — Displacement of Zn and Fe-S. (A) Zn atom could not displace the Fe-S cluster. Fifty µM RshA was reconstituted with the [Fe-S] cluster and then treated with equimolar and two molar excess of ZnCl2 for different time periods to estimate the affinity of protein with both the metals. % residual [Fe-S] cluster is based on the A400. (B) Displacement of zinc by [Fe-S] cluster in zinc saturated RshA protein. 50 µM RshA was saturated with Zn and then subjected for reconstitution with equimolar concentrations of Fe3+ and sulfide. The absorption scan profile confirms the presence of a [4Fe-4S] cluster and [Fe-S] could easily replace zinc. Note that RshA has more affinity to [Fe-S] than to zinc. All the absorption scans were observed between 300 and 800 nm. (TIF) [file pone.0043676.s002.tif]
